# Irregular breakfast eating and health status among adolescents in Taiwan

**DOI:** 10.1186/1471-2458-6-295

**Published:** 2006-12-07

**Authors:** Rea-Jeng Yang, Edward K Wang, Yeu-Sheng Hsieh, Mei-Yen Chen

**Affiliations:** 1 Nursing Department, National Taipei College of Nursing, Taipei, Taiwan; 2 Neurology Department, Tao-Yuan Veteran Hospital, Tao-Yuan, Taiwan; 3 Department of Agricultural Extension, National Taiwan University, Taipei, Taiwan; 4 Nursing Department, Chang Gung Institute of Technology, Wen-Hwa 1^st ^Road, Kwei-Shan, Tao-Yuan, Taiwan

## Abstract

**Background:**

Regular breakfast eating (RBE) is an important contributor to a healthy lifestyle and health status. The aims of the present study were to evaluate the relationships among irregular breakfast eating (IRBE), health status, and health promoting behavior (HPB) for Taiwanese adolescents.

**Methods:**

A cross-sectional, descriptive design was used to investigate a cluster sample of 1609 (7^th ^-12^th ^grade) adolescents located in the metropolitan Tao-Yuan area during the 2005 academic year. The main variables comprised breakfast eating pattern, body weight, and health promoting behaviors. Data were collected by a self-administered questionnaire.

**Results:**

A total of 1609 participants were studied, 64.1% in junior high school and 35.9% in high school, boys (47.1%) and girls (52.9%) ranging in age from 12–20 years. Of the total participant population, 28.8% were overweight and nearly one quarter (23.6%) reported eating breakfast irregularly during schooldays. The findings indicated that adolescents with RBE had a lower risk of overweight (OR for IRBE vs. RBE = 1.51, 95% CI: 1.12, 2.04), and that the odds of becoming overweight were 51% greater for IRBE than for RBE even after controlling for demographical and HPB variables. IRBE also was a strong indicator for HPB. However, the profile of the high-risk IRBE group was predominantly junior high schoolchildren and/or children living without both parents.

**Conclusion:**

This study provides valuable information about irregular breakfast eating among adolescents, which is associated with being overweight and with a low frequency of health promoting behavior. School and family health promotion strategies should be used to encourage all adolescents to eat breakfast regularly.

## Background

Regular breakfast eating (RBE) has been identified as an important factor in nutrition, especially during growth. Eating breakfast regularly is also an important contributor to a healthy lifestyle and health status. Several studies [1-4] have revealed that smoking, frequent alcohol used, and infrequent exercise were significantly associated with adolescent breakfast skipping. Moreover, many studies have shown significant relationships between skipping breakfast and depressive symptoms, stress, catching cold, chronic disease [5], and high body mass index (BMI) in adolescents [1,6-13]. Notably, Chen et al. [14] found that irregular breakfast eating (IRBE) had a significant negative association with quality of life among Japanese schoolchildren. Some studies [4,15] have shown that earlier menarche or dysmenorrheal is more common in girls with IRBE than in girls with RBE.

RBE has been shown to contribute significantly to children's daily nutrient intake and nutritional well-being and to affect the adequacy of their total daily intake [4,16]. IRBE or lack of breakfast has been implicated as a contributor to poor school performances: e.g. lower attendance rate; lower scores on math, English, social study and science; impaired short-term memory, problem solving skills and mental performance; and lower class rank [17-21].

Over the past decade, public health institutions around the world have placed increased emphasis on the importance of healthy lifestyles. Previous studies have shown that healthy lifestyles decrease susceptibility to disease and increase longevity [22]. Some studies on Taiwanese schoolchildren [23,24] have shown that the level of health status perception decreases with educational level, from elementary to college. Those students with a higher educational background exhibited the least health-promoting lifestyles; however, students with better health perceptions were found to exhibit better health-promoting behaviors [24]. The results of Chen et al. [23] also revealed that overweight adolescents had fewer health promoting behaviors than those who were not overweight. These findings strongly suggest that public health professionals need to take heed of what is happening to youth in Taiwan. Research has revealed that breakfast is the most important meal of the day. The aims of the present study were to evaluate the relationships among IRBE, health status, and health promoting behavior for Taiwanese adolescents. We hypothesized that IRBE is strongly related to being overweight and to negative health behaviors. We tested four hypotheses, focusing on Taiwanese adolescents, while controlling for the effects of potential confounders. The hypotheses were as follows. Hypothesis 1: there is a significantly positive association between IRBE and overweight status. After testing this main proposition, we clarified some confounding effects. Hypothesis 2: the positive effect of IRBE on overweight status is increased by lower frequency of health promoting behaviors. Hypothesis 3: there is a negative relationship between IRBE and HPB. Finally, we explored the characteristics of the group at high risk of IRBE.

## Methods

### Study design and population

A cross-sectional descriptive study design was used. This study was conducted at 10 public junior high schools or high schools in the Tao-Yuan metropolitan area, northern Taiwan. In total, there were 46 schools (about 85,000 students) distributed in the Tao-Yuan metropolitan area in 2005. Participants were enrolled in 6 junior high schools (7^th^–9^th ^grades) and 4 high schools (10^th^–12^th ^grades) during the 2005 academic year. We used a convenience sampling strategy for these schools. The participants were selected using simple cluster sampling by class. The study subjects were selected according to the following criteria: (1) the individual had no physical or mental handicap; (2) both guardian and adolescent were willing for the latter to participate in the study.

### Human subject protection and data collection procedures

The study was approved by the Chang Gung Memorial Hospital Review Board (CGMH, No 2004–6145). Informed written consent was obtained from the subjects and their guardian(s) after permission had been obtained from the relevant school administrators. Parents were informed about the survey and had an opportunity to review the questionnaire; that is, an invitation letter, emphasizing that the responses would be confidential, was sent together with the questionnaire to each student's guardian. The investigators explained the study to all the students. Students completed the questionnaire anonymously and used about 30 minutes to complete the scale. They could decline to participate in the project at any time while completing the questionnaire.

### Measures

Data were collected by a self-administered questionnaire. The questionnaire was composed as follows:

**Demographic characteristics **were gender; age; school level; parents' educational level; and living arrangements (whether the student lived with both parents, a single mother/father, grandparents or other relatives).

Regular breakfast eating (RBE) was defined as a meal taken before 9:00 a.m. each weekday (Monday to Friday). This time cutoff accords with discussions with school administrators and takes account of most school schedules in Taiwan. To obtain valid reporting of regular breakfast eating and to ensure the reliability and validity of measurements, the method was established in two steps. First, ten junior-high and nine high school adolescents were invited to participate in a pilot study to test content validity. None of these students appeared to have any doubt about their own assessment of regularity in eating breakfast. Next, all participants were instructed in how to count their breakfast attendances during the study period: for example, "irrespective of the content of breakfast, you treat the meal as your breakfast if it is eaten before 9:00 a.m." Finally, the students responded to a self-administered open-ended question: "Generally speaking, how many days did I eat breakfast between Monday and Friday?" These frequencies were recorded as "Irregular breakfast eating, IRBE" if the answer was "3 days or fewer", and as "Regular breakfast eating, RBE" if the answer was "four or five days".

**Health status **was determined by Body Mass Index (BMI). The height and weight were measured by self-report and the BMI was calculated by the standard formula: weight (kg) divided by height (m^2^). BMI was plotted on the age and sex-specific cutoff points to define the different body sizes of adolescents according to nationally accepted guidelines [25]. Each student was classified as overweight (>85th) or not overweight (>85th percentile) for age and sex. For example, in Taiwan, a 15-year-old boy with a BMI > 23.1 was defined as overweight, whereas a 15-year-old girl with a BMI > 22.7 was considered overweight.

**Health promoting behaviors **were measured by using the Health Promoting Behavior (HPB) scale [26], which is considered valid and reliable. The HPB comprises 40 items assessing six dimensions of behavior: (1) nutrition (eating 3 meals a day, five groups each meal, etc.); (2) social support (speaking to and sharing feelings with others, talking about personal problems with others, etc.); (3) life-appreciation (making an effort to like oneself, thinking positively, etc.); (4) health responsibility (tooth brushing after meals, observing one's body at least monthly, etc.); (5) stress-management (smiling or laughing every day, making schedules, setting priorities, etc.); and (6) exercise behavior (exercising rigorously for 30 minutes at least 3 times per week, performing stretching exercises daily, etc.). The measuring instrument used to obtain the frequency of reported behaviors was a self-reporting Likert scale with a five-point response format, "never, rarely, sometimes, usually, always", with the rating score ranging from 1 to 5.

### Statistical analysis

The risk (odds ratio) of overweight status and associated 95 percent confidence intervals was estimated using simple logistic regression analysis. Multivariable logistic regression modeling was used to control all risk estimates for covariates. Possible covariates, participants' health-promoting behaviors and demographic characteristics, including school level, age, gender, living arrangement, perceived health status, father's and mother's educational levels and the interaction terms of gender and school level, were evaluated as potential confounders of the relationship between eating patterns and overweight status.

## Results

### Description of the participants

Of the 1710 participants, 42 were excluded from the study owing to the absence of parental consent, and another 59 were removed for the following reasons: 52 had multiple invalid responses and 7 cited personal reasons for declining to participate. The valid response rate was 94.1%. Descriptive characteristics of the subjects (n = 1609) are given in Table 1. Briefly, 64.1% of the students were junior high school (grades 7–9), and 35.9% were high school (grades 10–12), aged from 12 to 20 years (14.99 ± 2.03); 47.1% were boys. A total of 85.1% of the participants lived with parents. The remaining 15% lived with a single parent or relatives such as grandparents, aunts or uncles. Most fathers and mothers of the participants had completed junior or high school education (77.3% and 85.3%, respectively).

Table 1 shows that 76.4% of the respondents regularly ate breakfast during schooldays and 23.6% of students were classified as IRBE; 28.8% of students were overweight; and 12.6% perceived their own health status as worse than that of their classmates. With respect to health promoting behaviors, the mean score of total HPB was 3.4 (135.20/40 = 3.4); the average scores of subscales ranged from 3.0 to 3.6. In general, participants responded to the frequency of their health behaviors as "sometimes".

### Relationships between IRBE and being overweight

Table 2 shows the risk of being overweight. Logistic regression was conducted and un-standardized coefficients are reported. Model 1 is a baseline model with IRBE as predictor. IRBE has significant effects on overweight status; odds ratio 1.66 (95% CI: 1.30–2.11). In model 2 of Table 2, we added controls for health-promoting behaviors (HPBs). The results show that the positive effect of IRBE on overweight status was increased by about 10% [(0.55–0.50)/0.50*100] when the effect of HPB was excluded from the model. In model 3 of Table 2, we added further controls for the demographic variables. The results revealed that IRBE remained strongly associated with overweight status after controlling for potential confounding factors, HPBs and demographics.

### Relationships between IRBE and HPB

Table 3 shows the relationships between IRBE and HPB. Model 1 of Table 3 revealed that IRBE and HPB were negatively associated (*p *< 0.0001) without control. However, IRBE and HPB retained a strongly negative association even when the effect of demographics were excluded (by controlling demographics), as seen in model 2 of Table 3.

### The profile of the high-risk group for IRBE

In Table 4, we summarize the characteristics of the group at high risk of IRBE. In model 1 of Table 4, individual characteristics were examined. The results showed that the school level was significantly associated with IRBE. In model 2 of Table 4, family characteristics were added. The results indicated that both the availability of parents and school level were strongly associated with IRBE. Participants living with both parents tended to have a lower risk of IRBE (odds ratio for living with parents vs. without = 0.64, 95% confidence interval: 0.47, 0.86). The results of model 2, table 4, show that parents' educational levels were not obviously associated with IRBE. However, the high school students had a lower prevalence of IRBE, as shown in models 1 and 2.

## Discussion

The results of the current study support the hypothesis that IRBE has a strong relationship with overweight status and health behaviors even after controlling for potential confounders, e.g. demographic and HPB variables. A higher overweight status risk was observed among participants reporting IRBE. Furthermore, IRBE was associated with worse HPB in these adolescents.

### Nearly one quarter of adolescents often skip breakfast

Overall, 23.6% of participants reported that they ate breakfast irregularly on schooldays. The prevalence of breakfast skipping among Taiwanese adolescents was higher than in other countries. For example, Nicklas et al. and Shaw [16,27] reported that 19% of American and 12% of Australian adolescents skipped breakfast. A Japanese study by Murata [28] showed that over 15% of schoolchildren (15–19 years old) do not eat breakfast. The author proposed a reasonable explanation for breakfast skipping among Japanese adolescents: because schoolchildren go on to junior high school and high school, they must prepare to pass an entrance examination. Therefore, the schoolchildren usually prepare for the entrance examination the night before school, so they are prone to excessive food intake at night and have a tendency to frequent fast-food outlets. This social context also was found in Taiwan.

### Irregular breakfast eating is positively associated with being overweight

The findings indicated that adolescents with RBE had a lower risk of being overweight (odds ratio for IRBE vs. RBE = 1.51, 95% CI: 1.12, 2.04), and that the odds of being overweight for IRBE are 51% greater than for RBE even after controlling demographical and HPB variables. Several other studies including Stockman et al. [29] and Berkey et al. [10] have yielded similar results, showing that inconsistent or irregular breakfast eating was significantly associated with being overweight.

### RBE is positively associated with quality of life

We suggested that RBE was a useful predictor for adolescent health-promoting behaviors. Our study revealed that health-promoting behaviors differ between the RBE and IRBE groups. In other words, the RBE adolescents had a higher frequency of health promoting behaviors, such as stress management (e.g. smiling or laughing), health responsibility (e.g. tooth brushing after meals), exercise, nutrition, social support and life appreciation behaviors than those who did not eat breakfast regularly. Some of these findings are consistent with previous studies [4,30,31]. For example, the study by Sjoberg et al. [4] on grade 9 (15–16y) students showed that irregular breakfast eating was related to negative lifestyle factors such as smoking and to irregular intake of lunch and dinner. These groups had significantly lower intakes of micronutrients but higher intakes of sucrose and alcohol compared to the groups with regular breakfast intake. Bruno-Ambrosius et al. [31] conducted a study of breakfast eating and tooth brushing in relation to dental caries in Sweden, and found that omission of breakfast was significantly associated with caries (decay). Smith [7] observed that eating breakfast is regularly associated with fewer colds and milder symptoms. Cartwright et al. [9] found that IRBE among adolescents was associated with greater stress. Fulkerson et al. [8] pointed out that depressive symptoms were negatively associated with eating breakfast. In addition, in Japan, Chen et al. [14] found that adolescents who seldom ate breakfast were more likely to have poor qualities of life. A possible explanation could be that skipping breakfast results in less energy in the morning and less motivation to participate in physical activity [2,3]. Physical activity among adolescents is associated with benefits in the prevention and control of emotional distress and with improved self-esteem [32]. However, less evidence was available to support this relationship between IRBE and quality of life. Further investigation of these associations by prospective studies is warranted.

### The characteristics of the group at high-risk of IRBE

Why do Taiwanese adolescents often skip breakfast? From the results there were two dominant factors, school level and living arrangements. First, junior high school students were vulnerable. The stage of development might provide an explanation. The impact of development on bio-psycho-sociology in early adolescents is perhaps greater than in late adolescents. Second, students without available parents were vulnerable, too. Videon et al. [33] also discovered that parental presence was associated with a lower risk of skipping breakfast. This might be a second reason why families do not regulate their children's eating habits, especially single-parent families, since 14.9% of the subjects lived without both parents. The findings also showed that adolescents living without both parents tended to skip breakfast often. In Taiwan, as elsewhere in the world, the enhancement of lifelong health promotion throughout society, beginning with youth and the family, is a key issue for the government. Therefore, further studies should attempt to educate adolescents, parents, teachers and schools about the importance of eating breakfast.

### Strengths and limitations

The first strength of this study was that the participants were drawn by cluster sampling from multiple settings. This allowed us to validate the data obtained. Furthermore, this study provides valuable information about breakfast skipping among Taiwanese adolescents and its disadvantages in respect of overweight status and health promoting behavior. This provided important recommendations for specific health promotion policies relating to our youth and involving the whole community.

The study has several limitations and these influence the generalizability of the findings. First, since the data were collected through a self-reporting measure, it is possible that the findings were affected by a social desirability response set. Second, breakfast eating was only counted from Monday to Friday; seven-day measurements or in-depth interviews might increase the reliability and validity of our understanding of this phenomenon. Third, we did not explore the content or quality of breakfast. Fourth, we did not explore the variety of ethnicities and household incomes. In addition, because we lack the ID for each school, examination of the IRBE cluster effect on overweight status could not proceed in this study. However, owing to the cross-sectional nature of this study, we cannot resolve the direction of the relationships among breakfast eating, health promoting behavior, overweight, and other variables measured in this study. More studies are expected in the future, not only considering variations in ethnicity, income and parental employment status, but also evaluating the effect of aggregation from hierarchical data.

## Conclusion

Despite these limitations, this study contributes to the literature on the impact of IRBE among Taiwanese adolescents in that (a) it shows that a high proportion of adolescents in Taiwan skip breakfast during schooldays, (b) skipping breakfast correlates with being overweight and with less health-promoting behavior, (c) the findings could be related to health strategies aimed at decreasing the barriers to promoting a healthy lifestyle among our youth. Schools have the potential to influence students' and families' attitudes regarding healthy breakfast and exercise. Since most of the adolescents spend a large portion of their day in school, modified school policies providing health education and healthy breakfast could help to prevent them skipping this important meal.

## Competing interests

The author(s) declare that they have no competing interests.

## Authors' contributions

RY: Statistic analysis, interpretation of results, writing the paper

EW: Co-analyzer, participating in revision of the paper

YH: Supervised the statistic analysis and interpretation of results, participating in revision of the paper

MC: Study design, data collection, data analysis, writing the paper

Authors have read and approved the final manuscript.

## Pre-publication history

The pre-publication history for this paper can be accessed here:



## Figures and Tables

**Table 1:** Demographic and descriptive characteristics (n = 1609)

Variables	n	%	Mean	SD
*Individual characteristics*				
Grader				
Junior high school	1038	64.1		
High school	582	35.9		
Age			14.99	2.03
Gender				
Boy	758	47.1		
Girl	850	52.9		
Perceived health status				
Better	318	19.8		
Average	1086	67.6		
Worse	203	12.6		
BMI			21.22	4.26
Overweight ^a^	464	28.8		
Non-overweight	1145	71.2		
				
*Family characteristics*				
Parents availability				
Living with parents	1369	85.1		
Living without parents	240	14.9		
Father's education levels				
High school and less				
College and above	1243	77.3		
Mother's education levels	364	22.7		
High school and less	1369	85.3		
College and above	235	14.7		
				
*Health promoting behaviors (HPB)*				
Breakfast eating pattern				
Regular breakfast eating (RBE)	1229	76.4		
Irregular breakfast eating (IRBE)	380	23.6		
Nutrition			20.93	3.95
Social support			25.32	4.98
Health responsibility			24.70	5.71
Life appreciation			28.47	6.04
Exercise behavior			14.85	4.01
Stress management			20.91	4.32
Total HPB score			135.20	21.88

^a^Overweight was defined as BMI > 85^th ^percentile specific for age and sex.

Note: The scale items are as follows: nutrition 6, social support 7, health responsibility 8, life appreciation 8, exercise behavior 5, stress management 6, and total HPB 40. Rated on a five-point scale; 5 = always; 4 = usually; 3 = sometimes; 2 = rarely; 1 = never.

**Table 2:** Association of irregular breakfast eating, health promoting behaviors and overweight status of adolescents

	Model 1 (n = 1609)			Model 2 (n =1590)			Model 3 (n = 1582)		
			
Independent variables	B^a^	OR	95% CI	B^a^	OR	95% CI	B^a^	OR	95% CI
IRBE (1 = IRBE)	0.50 ***	1.66	1.30-2.11	0.55 ***	1.74	1.30-2.33	0.42 ***	1.51	1.12-2.04
Health promotion behavior									
Nutrition				-0.05 **	0.95	0.91-0.99	0.03	1.03	0.99-1.07
Social support				0.03	1.03	0.99-1.06	0.01	1.00	0.97-1.04
Health responsibility				0.04 **	1.04	1.01-1.06	-0.02	0.98	0.96-1.01
Life appreciation				0.01	1.00	0.98-1.03	-0.01	1.00	0.97-1.03
Exercise behavior				0.01	1.01	0.97-1.04	-0.04 *	0.96	0.92-0.99
Stress management				0.02	1.02	0.98-1.05	-0.04	0.96	0.93-1.00
Control variables							b		

^a ^Unstandardized regression coefficients.

^b ^Controls include parents availability, parents' education level, gender, perceived health status, school level, and gender × school level.

*p < .05;

**p < .01; ***p < .001 (two-tailed tests).

**Table 3:** Association between irregular breakfast eating and health-promoting behavior

Dependent variables	Model 1			Model 2 (model 1 + Covariates)^b^		
		
Nutrition	B^a^		SE	B^a^		SE
Social support	-4.51	***	0.10	-4.54	***	0.20
Health responsibility	-2.46	***	0.29	-2.21	***	0.28
Life appreciation	-2.34	***	3.30	-2.23	***	0.33
Exercise behavior	-2.81	***	0.35	-2.62	***	0.34
Stress management	-0.91	***	0.24	-1.01	***	0.22
	-2.14	***	0.25	-2.20	***	0.24

Note: These regressions are conducted by regressing one promoting behavior on IRBE each time.

^a ^Unstandardized regression coefficients with standard errors.

^b ^Covariates include parent availability, parents' education level, gender, perceived health status, school level, and gender × school level.

***p <.001 (two-tailed tests).

**Table 4:** Association between demographic variables and irregular breakfast eating

Independent variables	Model 1 (n = 1606)			Model 2 (n = 1601)		
		
	B^a^	OR	95% CI	B^a^	OR	95% CI
Parents availability (1 = living with parents)				-0.45 **	0.64	0.47-0.86
Parent's education						
Father's education years (1 = college and above level)				-0.02	0.98	0.71-1.36
Mother's education years (1 = college and above level)				0.02	1.02	0.69-1.51
Individual characteristics						
Gender (1 = male)	0.15	1.16	0.88-1.53	0.15	1.16	0.88-1.54
Perceived health status	-0.11	0.90	0.73-1.11	-0.10	0.90	0.73-1.11
School level (1 = high school)	-0.38 *	0.68	0.49-0.96	-0.38 *	0.69	0.49-0.97
Gender × school level	-0.03	0.98	0.59-1.61	-0.01	0.99	0.60-1.64

^a ^Unstandardized regression coefficients.

*p <.05; **p <.01
